# Strip-width determines competitive strengths and grain yields of intercrop species in relay intercropping system

**DOI:** 10.1038/s41598-020-78719-y

**Published:** 2020-12-14

**Authors:** Muhammad Ali Raza, Liang Cui, Ruijun Qin, Feng Yang, Wenyu Yang

**Affiliations:** 1 College of Agronomy, Sichuan Agricultural University, Chengdu, China; 2 Sichuan Engineering Research Center for Crop Strip Intercropping System, Chengdu, China; 3 Key Laboratory of Crop Ecophysiology and Farming System in Southwest China, Chengdu, China; 4 Crop Research Institute, Liaoning Academy of Agricultural Sciences, Liaoning, China; 5 Hermiston Agricultural Research and Extension Center, Oregon State University, Hermiston, USA

**Keywords:** Plant sciences, Environmental sciences

## Abstract

Maize/soybean relay intercropping system (MSR) is a popular cultivation method to obtain high yields of both crops with reduced inputs. However, in MSR, the effects of different strip widths on competitive strengths and grain yields of intercrop species are still unclear. Therefore, in a two-year field experiment, soybean was relay-intercropped with maize in three different strip-width arrangements (narrow-strips, 180 cm; medium-strips, 200 cm; and wide-strips, 220 cm), and all intercropping results were compared with sole maize (SM) and sole soybean (SS). Results showed that the optimum strip-width for obtaining high grain yields of maize and soybean was 200 cm (medium-strips), which improved the competitive-ability of soybean by maintaining the competitive-ability of maize in MSR. On average, maize and soybean produced 98% and 77% of SM and SS yield, respectively, in medium-strips. The improved grain yields of intercrop species in medium-strips increased the total grain yield of MSR by 15% and land equivalent ratio by 22%, which enhanced the net-income of medium-strips (by 99%, from 620 US $ ha^−1^ in wide-strips to 1233 US $ ha^−1^ in medium-strips). Overall, these findings imply that following the optimum strip-width in MSR, i. e., strip-width of 200 cm, grain yields, and competitive interactions of intercrop species can be improved.

## Introduction

**Figure 1:**
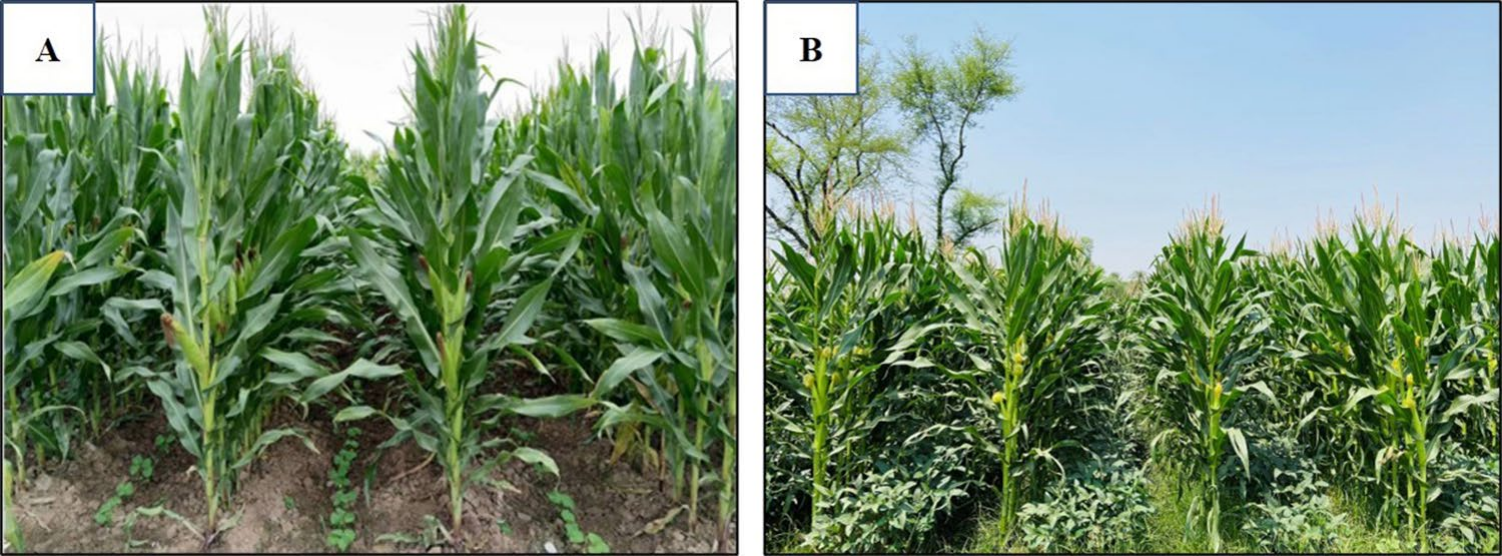
Example of traditional intercropping systems. (**A**) maize soybean relay-intercropping system (Photo: Muhammad Ali Raza, location: Sichuan province, China); (**B**) maize soybean intercropping systems (Photo: Muhammad Ali Raza, location: Punjab province, Pakistan).

Intercropping or mixed cropping systems are mostly practiced with a low degree of mechanization, especially in developing countries^1^. Therefore, these cropping systems are under pressure due to the scarcity of rural labor and low labor income from farming activities^2^. Mechanization can increase the total profit of these systems, and it could be a solution to the less availability of rural labor for farming activities. However, for mechanization, we need to change or replace the current intercropping designs, which are in practice by farmers (e.g., Fig. 1). For instance, strip intercropping systems can be used with optimum-strip widths (i.e., wider-strips could be designed that are wide enough for the use of machinery). Nevertheless, the use of wider-strips in intercropping systems will raise the question of whether strip intercropping systems are advantageous over sole cropping systems or not? Therefore, it is important to determine the optimum-strip width, which can increase the crop yields of intercrop species and provide enough space for the use of machines in intercropping systems.

Generally, in intercropping systems, two crops are planted at an appropriate row spacing, allowing positive interactions between different species. These include the benefits of mutual interactions between adjacent allospecific plants, e. g., facilitation in water and nutrient uptake, which are maximized^3,4^. Several studies tested wider-strip widths and revealed that intercropping systems with wider-strips of 3 m^5^, 3.1 m^6^, 3.3 m^7^, 3.4 m^8^, and 6 m^9^ achieved the highest LER compared to narrow-strips. Therefore, intercropping of crops with larger operating strips would be easier to manage with existing farm machinery, and it will produce high profits for farmers. In contrast, recent research revealed that the optimum-strip width to obtain the maximum benefits of intercropping is 1 m, and if the strip width increased from 1 m, the benefits of intercropping decreased^10,11^. Similarly, the land equivalent ratio (LER) of maize/common bean intercropping system was decreased from 1.55 to 1.27 when the row ratio was increased from 2:2 (two rows of maize with two rows of common bean) to 5:5 (five rows of maize with five rows of common bean), as strip width increased^12^. These results reinforce the concerns mentioned above that intercropping systems will become less attractive as the local farming community in developing countries want to replace labor with machinery for that they need larger operating strip widths.

Among intercropping systems, cereal-legume intercropping has been regarded as an important and advantageous combination^13^. It has high potential to reduce the inputs, improve environmental quality and decrease nutrient emission^14^, particularly in developing countries (e. g. China, Pakistan, and India) where agriculture is characterized by the use of high fertilizer inputs and high nutrient emissions (i.e., nitrogen oxides) to the atmosphere^15,16^. Therefore, there is an increasing interest from researchers and policymakers for including legumes (crops which can fix atmospheric nitrogen such as soybean) in the current cropping systems to reduce external inputs and boost natural fixation of nitrogen^17,18^. Such advantages of cereal-legume intercropping can be achieved through a maize/soybean relay intercropping system, especially in those areas where the growing season is too short for double cropping^19^. The maize/soybean relay intercropping system (MSR) is the best intercropping system in yield stability^20^, resource utilization^21^, and economic profit^22^. In MSR, maize yield is often equal or sometimes higher than the sole maize yield^23^. This system improves the soil quality^14^, and increases the availability of essential plant nutrients, i.e., nitrogen and phosphorus^24,25^.

Nevertheless, tradeoffs between intercrop species exist in MSR^22,26,27^. In many past reports, scientists have reported that soybean suffers from heavy maize shading, especially during the co-growth phase^13^. Maize shading adversely affects the morphology (plant height and stem diameter), physiology (photosynthesis, chlorophyll structure, and concentration), and biochemistry (activities of antioxidants, sucrose, and starch accumulation) of intercropped soybean plants in MSR^28–30^. Therefore, intercropped soybean produces a lower yield in MSR as compared to sole soybean yield^13,31^. However, we may not only reduce the adverse effects of maize shading on soybean but also need to provide enough space for machinery by increasing inter-row spacing in MSR, which will also increase the total strip width of the maize/soybean intercropping system. Therefore, a comprehensive study is required to determine the optimum spacing between the rows of soybean and maize in this system, which can increase the benefits of intercropping by increasing beneficial interspecific interactions (facilitation) and decreasing detrimental interspecific interactions (competition).

Thus, we conducted a two-year field experiment to study the light environment, photosynthesis, dry matter accumulation, yield, and yield components of soybean in sole cropping and relay intercropping systems. We aimed to determine the response to changing the inter-row spacing in MSR for soybean and hypothesized that: (i) soybean produces a lower grain yield when grown in narrow-strips under MSR; (ii) increasing the inter-row spacing in MSR would increase the grain yield of soybean without affecting the maize grain yield, and (iii) the wider-strips would be more productive and beneficial in increasing the benefits of intercropping systems than narrow-strips.

## Results

### Photosynthetically active radiation transmittance

The PAR-transmittance of SS, T_1_, T_2_, and T_3_ is shown in Table 1. At all sampling stages (V5, V7, and R1), the average values for PAR-transmittance revealed that different inter-row spacing treatments had a significant (*P* < 0.05) effect on the PAR-transmittance of soybean in MSR. The maximum PAR-transmittance of soybean was always noted in SS than the corresponding values in T_1_, T_2_, and T_3_ under MSR. However, the PAR-transmittance of soybean increased with the increase in inter-row spacing in MSR. At R1, average over the two years, the highest PAR-transmittance (57%) was noticed under treatment T_3_, while the lowest PAR-transmittance (31%) was calculated in T_1_. Furthermore, the PAR of soybean plants at all stages (V5, V7, and R1) followed the same pattern, with a trend of SS > T_3_ > T_2_ > T_1_, suggesting that PAR-transmittance of understory crops in intercropping systems was closely associated with the changes of inter-row spacing.

**Table 1:** Effect of different strip-width treatments on photosynthetically active radiation transmittance (PAR-transmittance) of soybean canopy at the fifth trifoliate stage (V5), seventh trifoliate stage (V7), and flower initiation stage (R1) in cropping seasons from 2012 to 2013.

Years	Treatments	PAR-transmittance (%)		
		V5	V7	R1
2012	T_1_	63.4 ± 2.3c	45.4 ± 3.4c	31.9 ± 2.4c
	T_2_	82.3 ± 1.9b	67.8 ± 3.3b	54.9 ± 1.3b
	T_3_	84.3 ± 3.8b	69.6 ± 2.9b	59.0 ± 1.3b
	SS	100.0 ± 0.0a	100.0 ± 0.0a	100.0 ± 0.0a
	LSD (0.05)	6.31	5.53	4.53
2013	T_1_	61.6 ± 2.3d	43.9 ± 2.1c	30.1 ± 1.6c
	T_2_	79.2 ± 2.2c	66.1 ± 1.4b	53.1 ± 1.6b
	T_3_	84.9 ± 1.5b	67.9 ± 1.8b	55.7 ± 1.5b
	SS	100.0 ± 0.0a	100.0 ± 0.0a	100.0 ± 0.0a
	LSD (0.05)	5.67	3.21	5.52

Treatment codes represent narrow-strips (T_1_, 180 cm); medium-strips (T_2_, 200 cm); and wide-strips (T_3_, 220 cm) in maize/soybean relay intercropping system. SS is the sole cropping system of soybean. Means are averages over three replicates ± standard error of the mean. Means that do not share the same letters in a column differ significantly at p ≤ 0.05 using least significant differences, calculated separately for each year.

### Leaf area index and dry matter

At all sampling stages, soybean in SS achieved the maximum values for the leaf area index compared to other treatments (Table 2). However, the leaf area index of soybean was significantly (*P* < 0.05) increased with increasing the inter-row spacing in MSR. In MSR treatments, the maximum (0.5, 1.6, and 2.3 at V5, V7, and R1, respectively) leaf area index of soybean was recorded in treatment T_3_, whereas the minimum (0.3, 1.2, and 1.9 at V5, V7, and R1, respectively) leaf area index of soybean was measured in T_1_.

**Table 2:** Effect of different strip-width treatments on leaf area index of soybean at fifth trifoliate stage (V5), seventh trifoliate stage (V7), and flower initiation stage (R1) in cropping seasons from 2012 to 2013.

Years	Treatments	Leaf area index		
		V5	V7	R1
2012	T_1_	0.36 ± 0.01c	1.23 ± 0.05c	1.84 ± 0.05c
	T_2_	0.56 ± 0.02b	1.69 ± 0.01b	2.39 ± 0.01b
	T_3_	0.54 ± 0.02b	1.65 ± 0.02b	2.31 ± 0.01b
	SS	0.69 ± 0.01a	1.92 ± 0.02a	2.51 ± 0.03a
	LSD (0.05)	0.06	1.12	0.11
2013	T_1_	0.33 ± 0.01c	1.25 ± 0.05c	1.92 ± 0.04c
	T_2_	0.54 ± 0.01b	1.73 ± 0.02b	2.42 ± 0.02b
	T_3_	0.51 ± 0.01b	1.63 ± 0.01b	2.33 ± 0.03b
	SS	0.64 ± 0.02a	2.04 ± 0.05a	2.62 ± 0.01a
	LSD (0.05)	0.05	0.10	0.09

Treatment codes represent narrow-strips (T_1_, 180 cm); medium-strips (T_2_, 200 cm); and wide-strips (T_3_, 220 cm) in maize/soybean relay intercropping system. SS is the sole cropping system of soybean. Means are averages over three replicates ± standard error of the mean. Means that do not share the same letters in a column differ significantly at p ≤ 0.05 using least significant differences, calculated separately for each year.

All treatments (SS, T_1_, T_2_, and T_3_) significantly (*P* < 0.05) changed the dry matter of soybean plants (Table 3). Average across the years, treatment SS produced the highest (1.2 g plants^−1^, 5.0 g plants^−1^, 12.2 g plants^−1^) dry matter of soybean at V5, V7, and R1, respectively, while soybean accumulated lowest (0.3 g plants^−1^, 1.2 g plants^−1^, 2.5 g plants^−1^) dry matter at V5, V7, and R1, respectively in T_1_ under MSR. Overall, dry matter accumulation of soybean exhibited the trend SS > T_2_ > T_3_ > T_1_, indicating that increasing inter-row spacing between soybean and maize in MSR improved dry matter accumulation in soybean by decreasing the adverse impacts of maize shading on soybean. For example, averaged across the years, at R1, treatment T_2_ increased the dry matter of soybean by 131% compared to treatment T_1_ (Table 3).

**Table 3:** Effect of different strip-width treatments on dry matter of soybean at fifth trifoliate stage (V5), seventh trifoliate stage (V7), and flower initiation stage (R1) in cropping seasons from 2012 to 2013.

Years	Treatments	Dry matter (g plant^−1^)		
		V5	V7	R1
2012	T_1_	0.27 ± 0.01b	0.68 ± 0.08b	0.88 ± 0.09c
	T_2_	0.43 ± 0.02b	1.90 ± 0.22b	2.82 ± 0.55b
	T_3_	0.31 ± 0.01b	0.83 ± 0.10b	2.40 ± 0.49b
	SS	1.27 ± 0.18a	3.85 ± 0.68a	9.51 ± 0.65a
	LSD (0.05)	0.32	1.30	1.24
2013	T_1_	0.40 ± 0.01b	1.75 ± 0.07c	4.14 ± 0.32c
	T_2_	0.57 ± 0.05b	2.97 ± 0.17bc	8.78 ± 1.15b
	T_3_	0.50 ± 0.01b	2.52 ± 0.18b	7.38 ± 0.61b
	SS	1.12 ± 0.09a	6.22 ± 0.57a	14.88 ± 1.56a
	LSD (0.05)	0.20	1.09	3.01

Treatment codes represent narrow-strips (T_1_, 180 cm); medium-strips (T_2_, 200 cm); and wide-strips (T_3_, 220 cm) in maize/soybean relay intercropping system. SS is the sole cropping system of soybean. Means are averages over three replicates ± standard error of the mean. Means that do not share the same letters in a column differ significantly at p ≤ 0.05 using least significant differences, calculated separately for each year.

### Photosynthesis

The *Pn*, *Tr*, and *Gs* in SS were found significantly (*P* < 0.05) higher than other treatments in MSR (Table 4). However, all photosynthetic parameters (*Pn, Tr,* and *Gs*) except *Ci* of soybean leaves improved with the improvement in PAR-transmittance at soybean canopy in MSR. At V5, V7, and R1, the mean maximum *Pn* (11.3 μmol CO_2_ m^−2^ s^−1^, 14.1 μmol CO_2_ m^−2^ s^−1^, and 17.3 μmol CO_2_ m^−2^ s^−1^), *Tr* (4.8 mmol H_2_O m^−2^ s^−1^, 6.0 mmol H_2_O m^−2^ s^−1^, and 6.9 mmol H_2_O m^−2^ s^−1^), and *Gs* (0.6 μmol CO_2_ m^−2^ s^−1^, 0.7 μmol CO_2_ m^−2^ s^−1^, and 0.8 μmol CO_2_ m^−2^ s^−1^), respectively were measured in treatment T_2_, and the mean minimum *Pn, Tr,* and *Gs* values were observed under T_1_ in both years of study. Whereas, the average highest and lowest *Ci* values at all sampling stages were determined under treatment T_1_ and SS, respectively (Table 4).

**Table 4:** Effect of different strip-width treatments on photosynthetic characteristics of soybean at fifth trifoliate stage (V5), seventh trifoliate stage (V7), and flower initiation stage (R1) in cropping seasons from 2012 to 2013.

Years	Treatments	Photosynthetic Rate			Transpiration Rate			Stomatal Conductance			Intercellular CO_2_ Concentration		
		(μmol CO_2_ m^−2^ s^−1^)			(mmol H_2_O m^−2^ s^−1^)			(mol H_2_O m^−2^ s^−1^)			(μmol CO_2_ m^−2^ s^−1^)		
		V5	V7	R1	V5	V7	R1	V5	V7	R1	V5	V7	R1
2012	T_1_	6.9 ± 0.1c	8.4 ± 0.1c	11.1 ± 0.1d	3.3 ± 0.0d	3.6 ± 0.0d	5.0 ± 0.0d	0.3 ± 0.0c	0.5 ± 0.0d	0.6 ± 0.0c	285.0 ± 1.0a	309.0 ± 1.3a	321.9 ± 1.9a
	T_2_	11.2 ± 0.1b	14.1 ± 0.2b	17.7 ± 0.3c	4.9 ± 0.1c	6.0 ± 0.1c	6.9 ± 0.1c	0.6 ± 0.0b	0.7 ± 0.0c	0.8 ± 0.0b	246.4 ± 1.7b	264.7 ± 1.6b	274.0 ± 1.5b
	T_3_	11.1 ± 0.1b	13.9 ± 0.2b	16.9 ± 0.3b	4.7 ± 0.0b	5.8 ± 0.0b	6.7 ± 0.1b	0.5 ± 0.0b	0.6 ± 0.0b	0.8 ± 0.0b	243.5 ± 2.6b	261.4 ± 4.0b	271.9 ± 1.5b
	SS	14.0 ± 0.1a	16.7 ± 0.2a	20.6 ± 0.5a	6.0 ± 0.0a	6.5 ± 0.1a	8.5 ± 0.0a	0.7 ± 0.0a	0.8 ± 0.0a	0.9 ± 0.0a	214.3 ± 2.4c	228.6 ± 2.1c	252.7 ± 1.9c
	LSD (0.05)	0.38	0.56	0.75	0.15	0.16	0.22	0.07	0.03	0.05	7.29	4.83	6.73
2013	T_1_	6.8 ± 0.1c	8.4 ± 0.1c	10.4 ± 0.4c	3.1 ± 0.0c	3.7 ± 0.1c	4.7 ± 0.1c	0.3 ± 0.0c	0.5 ± 0.0c	0.5 ± 0.0c	289.3 ± 1.4a	313.0 ± 4.2a	323.4 ± 3.6a
	T_2_	11.3 ± 0.1b	14.0 ± 0.4b	17.0 ± 0.2b	4.8 ± 0.0c	6.0 ± 0.0b	6.8 ± 0.1b	0.6 ± 0.0b	0.7 ± 0.0b	0.8 ± 0.0b	249.2 ± 1.8b	267.2 ± 2.0b	265.7 ± 1.7b
	T_3_	11.0 ± 0.1b	14.0 ± 0.3b	16.9 ± 0.3b	4.7 ± 0.0b	6.0 ± 0.1b	6.6 ± 0.1b	0.5 ± 0.0b	0.7 ± 0.0b	0.7 ± 0.0b	244.0 ± 1.9b	265.0 ± 1.3b	269.4 ± 2.0b
	SS	14.0 ± 0.2a	17.0 ± 0.2a	21.2 ± 0.5a	5.9 ± 0.0a	6.7 ± 0.1a	8.3 ± 0.1a	0.7 ± 0.0a	0.8 ± 0.0a	0.9 ± 0.0a	219.5 ± 1.9c	231.6 ± 1.3c	252.5 ± 1.6c
	LSD (0.05)	0.51	0.76	1.09	0.09	0.24	0.37	0.07	0.06	0.05	6.33	8.98	9.08

Treatment codes represent narrow-strips (T_1_, 180 cm); medium-strips (T_2_, 200 cm); and wide-strips (T_3_, 220 cm) in maize/soybean relay intercropping system. SS is the sole cropping system of soybean. Means are averages over three replicates ± standard error of the mean. Means that do not share the same letters in a column differ significantly at p ≤ 0.05 using least significant differences, calculated separately for each year.

### Grain yield

Different inter-row spacing treatments (T_1_, T_2_, and T_3_) significantly (*P* < 0.05) affected the grain number (plant^−1^), grain weight (mg), and grain yield (kg ha^−1^) of soybean and maize in MSR, and both crops always produced the maximum grain number, grain weight, and grain yield in SS and SM (Table 5). However, among T_1_, T_2_, and T_3_, the average highest grain number (79.9 plant^−1^ and 522 plant^−1^) of soybean and maize was recorded in T_2_ and T_1_, respectively; while the mean maximum (206.8 mg and 230.3 mg) seed weight of soybean and maize was noticed in T_2_ treatment.

**Table 5:** Effect of different strip-width treatments on grain number, grain weight, grain yields of maize and soybean, and the total grain yield of maize/soybean relay intercropping system in cropping seasons from 2012 to 2013.

Years	Treatments	Grain number (plant^−1^)		Grain weight (mg)		Grain yield (kg ha^−1^)		Total grain yield
		Maize	Soybean	Maize	Soybean	Maize	Soybean	(kg ha^−1^)
2012	T_1_	593.4 ± 11.5a	51.9 ± 1.6c	226.0 ± 6.1b	206.8 ± 3.1 ^NS^	8406.3 ± 282.5a	735.7 ± 22.2d	9142.0 ± 261.4b
	T_2_	585.0 ± 11.1a	70.2 ± 2.1b	234.0 ± 4.9a	210.8 ± 5.9	8321.5 ± 26.5a	1292.1 ± 73.0b	9613.7 ± 60.4a
	T_3_	544.4 ± 12.6b	58.4 ± 1.4c	235.0 ± 7.7a	214.6 ± 5.2	7409.5 ± 122.8b	1058.2 ± 19.6c	8467.8 ± 105.7c
	SS	–	108.1 ± 2.1a	–	220.1 ± 4.5	–	1958.7 ± 80.1a	–
	SM	594.4 ± 15.1a	–	234.0 ± 4.7a	–	8437.7 ± 165.8a	–	–
	LSD (0.05)	27.26	6.59	4.74	19.12	434.18	210.85	423.19
2013	T_1_	451.4 ± 7.7 ^NS^	71.7 ± 2.0d	222.3 ± 9.6 ^NS^	200.1 ± 3.9 ^NS^	5799.0 ± 67.0a	1062.8 ± 5.7d	6861.8 ± 61.4a
	T_2_	449.7 ± 10.8	89.0 ± 1.6b	226.7 ± 8.3	202.9 ± 6.8	5870.0 ± 78.5a	1375.1 ± 34.6b	7245.1 ± 103.8a
	T_3_	429.1 ± 6.7	76.9 ± 2.9c	222.8 ± 5.4	197.6 ± 3.6	5227.0 ± 147.3b	918.9 ± 17.3c	6145.9 ± 155.5b
	SS	–	127.2 ± 2.5a	–	206.9 ± 6.1	–	1521.0 ± 25.8a	–
	SM	460.9 ± 13.2	–	226.2 ± 15.4	–	6069.5 ± 63.4a	–	–
	LSD (0.05)	38.67	2.47	34.73	15.86	354.16	73.30	448.43

Treatment codes represent narrow-strips (T_1_, 180 cm); medium-strips (T_2_, 200 cm); and wide-strips (T_3_, 220 cm) in maize/soybean relay intercropping system. SS is the sole cropping system of soybean, and SM is the sole cropping system of maize. Means are averages over three replicates ± standard error of the mean. Means that do not share the same letters in a column differ significantly at p ≤ 0.05 using least significant differences, calculated separately for each year.

*NS* non-significant.

In this study, the average across the years, relay-cropped soybean achieved 52% in T_1_, 77% in T_2_, and 57% in T_3_ of sole soybean grain yield, and relay-cropped maize achieved 98% in T_1_, 98% in T_2_, and 87% in T_3_ of sole maize grain yield. The grain yield of soybean exhibited the trend SS > T_2_ > T_3_ > T_1_. The grain yield of maize demonstrated the trend SM > T_1_ ≥ T_2_ > T_3._ Furthermore, the mean maximum (8429.4 kg ha^−1^) and minimum (7306.8 kg ha^−1^) total grain yield (soybean grain yield + maize grain yield) of maize/soybean relay intercropping system were obtained in treatments T_2_ and T_3_, respectively. Overall, the total grain yield of maize/soybean relay intercropping system showed the trend T_2_ > T_1_ > T_3_, suggesting that grain yields of both intercrop species were maximized when both crops complement each other with an optimum inter-row spacing (i. e., T_2_) between soybean and maize rows in MSR (Table 5).

### Land equivalent ratio and competition ratio

The mean land equivalent ratio values in T_1_, T_2_, and T_3_ treatments in maize/soybean relay intercropping system were higher than the one regardless of inter-row spacing treatments, indicating the yield and land advantage over sole maize and sole soybean (Table 6). Among all inter-row spacing treatments in MSR, the maximum LER was 1.76 in the T_2_, whereas the minimum LER was 1.44 in the T_3_, across two years. Among the three MSR treatments, the LER was highest at the inter-row spacing of 60 cm (T_2_) (Table 6).

**Table 6:** Effect of different strip-width treatments on competition ratios and land equivalent ratios (LER) of maize and soybean in cropping seasons from 2012 to 2013.

Years	Treatments	Competition ratio (%)		Land equivalent ratio		Total LER
		Maize	Soybean	Maize	Soybean	
2012	T_1_	2.65 ± 0.07a	0.38 ± 0.01b	1.00 ± 0.02 ^NS^	0.38 ± 0.01c	1.37 ± 0.03b
	T_2_	1.51 ± 0.16b	0.67 ± 0.06a	0.99 ± 0.02	0.66 ± 0.06a	1.65 ± 0.05a
	T_3_	1.62 ± 0.05b	0.62 ± 0.02a	0.88 ± 0.01	0.54 ± 0.01b	1.42 ± 0.01b
	LSD (0.05)	0.41	0.15	0.14	0.11	0.08
2013	T_1_	1.37 ± 0.02a	0.73 ± 0.01b	0.96 ± 0.01 ^NS^	0.70 ± 0.01c	1.65 ± 0.01b
	T_2_	1.07 ± 0.04b	0.94 ± 0.04a	0.97 ± 0.02	0.90 ± 0.02b	1.87 ± 0.02a
	T_3_	1.43 ± 0.06a	0.70 ± 0.03b	0.86 ± 0.03	0.60 ± 0.01a	1.47 ± 0.03c
	LSD (0.05)	0.14	0.08	0.13	0.09	0.16

Treatment codes represent narrow-strips (T_1_, 180 cm); medium-strips (T_2_, 200 cm); and wide-strips (T_3_, 220 cm) in maize/soybean relay intercropping system. Means are averages over three replicates ± standard error of the mean. Means that do not share the same letters in a column differ significantly at p ≤ 0.05 using least significant differences, calculated separately for each year.

*NS* non-significant.

Similarly, the competition ratio values of intercrop species followed the same trend with LER values of T_1_, T_2_, and T_3_ treatments in MSR (Table 6). Averaged across the years, the competition ratio values of maize (2.01 in T_1_, 1.29 in T_2_, and 1.53 in T_3_) were found higher than the competition ratio values of soybean (0.55 in T_1_, 0.81 in T_2_, and 0.66 in T_3_). However, with the medium inter-row spacing (i. e., T_2_), the competition ratio value of soybean plants significantly increased; for example, the competition ratio values of soybean were improved by 45% and 22% in T_2_ treatment compared to T_1_ and T_3_, respectively, suggesting the complementarity effect between maize and soybean in medium-strips.

### Economic analysis

Economic analysis for soybean and maize production under maize/soybean relay intercropping systems and sole cropping systems is shown in Table 7. The highest total income (1593 US $ ha^−1^ in 2012 and 873 US $ ha^−1^ in 2013) was obtained in treatment T_2_ under MSR, while the lowest total income (521 US $ ha^−1^ in 2012 and 92 US $ ha^−1^ in 2013) was noted in SS treatment. Overall, average over the years, the total income was enhanced by 54% and 99% in T_2_, compared to T_1_ and T_3_, respectively.

**Table 7:** Economic analysis for the effects of different strip width treatments on maize and soybean production in 2012 and 2013.

Treatments	Total expenditure (US $ ha^−1^)		Gross income (US $ ha^−1^)		Net income (US $ ha^−1^)		Average
	2012	2013	2012	2013	2012	2013	
T_1_	2820	2903	3402	3592	583	689	636
T_2_	2820	2903	3971	4015	1152	1112	1132
T_3_	2820	2903	3509	3667	690	764	727
SS	1725	1776	2396	2249	671	472	572
SM	1696	1747	2296	2316	599	569	584

Treatment codes represent narrow-strips (T1, 180 cm); medium-strips (T2, 200 cm); and wide-strips (T3, 220 cm) in maize/soybean relay intercropping system. SS is the sole cropping system of soybean, and SM is the sole cropping system of maize. The exchange rate of the per US dollar in 2012 and 2013 was 6.23 and 6.05 Chinese RMB (Yuan), respectively.

## Discussion

The main objective of this study was to test three hypotheses: the first hypothesis (soybean produces a lower grain yield when grown in narrow-strips under MSR) was strongly confirmed by our data. Soybean grain yield was significantly lower in narrow-strips (T_1_) than medium- (T_2_) and wider-strips (T_3_) under MSR. The second hypothesis (increasing inter-row spacing in MSR would increase the grain yield of soybean without affecting the maize grain yield) was partially confirmed by the data. Compared to narrow-strips in MSR, the medium-strips had a significantly higher soybean grain yield, and it did not decrease the maize grain yield, while the wider-strips produced significantly lower soybean and maize grain yield in MSR. The third hypothesis (the wider-strips would be more productive and beneficial in increasing the benefits of intercropping systems than narrow-strips) was also partially confirmed by the data. Relative to narrow-strips in MSR, both intercrops showed more complementarity effect in medium-strips, which ultimately enhanced the land productivity (high LER value) and total income of the system, whereas the wider-strips significantly reduced the intercropping benefits by decreasing the LER value and total income of MSR. Overall, these results suggest that wider-strips are not favorable for obtaining maximum benefits of intercropping systems.

### Photosynthetically active radiation transmittance

In this study, the leaf area index, photosynthetic rate, and dry matter accumulation of soybean were significantly affected by changing the inter-row spacing in MSR, which might be directly linked with the availability of PAR-transmittance at soybean canopy, because soybean is a C3 crop and sensitive to shade^23^. In all inter-row spacing treatments in MSR, maize intercepted more solar radiation due to its higher leaf area and height compared to soybean during the co-growth phase^26^, which significantly enhanced the competitive ability of maize. However, increasing the inter-row spacing from 50 to 60 cm (T_2_) significantly improved the leaf area index, photosynthetic rate, and dry matter accumulation of soybean, suggesting that the PAR-transmittance of relay-cropped soybean is dependent on the inter-row spacing in MSR. Another possible reason for this improvement in PAR-transmittance of soybean canopy is that maize foliage in T_2_ treatment occupied less row space, leaving a wide-open space for PAR to penetrate at soybean canopy, while in T_1,_ maize leaves filled the most of the row space, leaving a narrow opening for PAR-transmittance at soybean canopy. Consequently, the soybean plants received more PAR-transmittance in T_2_ compared to that of in treatment T_1_. These results are in agreement with the previous findings in which researchers have revealed that the spacing of 60 cm between the rows of intercrop species significantly reduced the maize shade by allowing more sunlight to penetrate to the soil surface^22^. Overall, these results exhibit that the selection of appropriate inter-row spacing is critical in improving the PAR-transmittance of soybean in MSR.

### Land use advantages

In relay intercropping systems, component crops have a comparatively short co-growth phase than in intercropping systems^32^. In these systems, interspecific-interactions during the co-growth phase determine the recovery growth and yield of late-maturing crops^33^. The medium inter-row spacing in MSR reduced the maize shade on the soybean canopy. It improved the initial growth and development of relay-cropped soybean in MSR, demonstrating the enhanced competitive ability of soybean by maintaining the competitive ability of maize in T_2_ than T_1_ and T_3_ treatments. This improved ability could help soybean to reduce the adverse impacts of interspecific competition for light^23^, water^4^, and nutrients^19^ in MSR. For instance, Raza et al. (2019) estimated that soybean accumulated 26% higher total major-nutrients in the medium inter-row spacing system than the narrow inter-row spacing system of MSR, explaining the reason for the soybean yield gain of the T_2_ system over T_1_ system^18^. Furthermore, compared to narrow-strips (T_1_), the extra growing space of 10 cm on both sides of the soybean rows with the optimum plant to plant spacing leads to a temporal niche differentiation, which relaxes competitive interactions, especially during the co-growth phase in MSR. However, in wider-strips (T_3_), the intra-specific competition for available resources increased due to the reduced plant to plant spacing among soybean and maize plants, which resulted in a disadvantage of yield and land equivalent ratio in MSR. Additionally, a reduced distance between soybean plants in wider-strips increased mutual shading within soybean plants, which in turn increased the lodging rate and decreased the seed yield of soybean plants, especially under the intercropping systems. Another possible reason for lower grain yield and competitive ability of intercrop species in wider strips was related to an apparent lack of complementarity between soybean and maize in MSR, partially caused by the complete temporal overlap of intercrop species, which results in intense intra-specific competition between soybean and maize and limited opportunity for complimentary resource capture in time. These results are in line with the results of wheat/chickpea intercropping^34^, maize/soybean intercropping^35^, and maize/common bean intercropping systems^12^, where the yields of intercrop species and intercropping advantages decreased as the strips become wider^11^.

Interspecific interactions (above- or below-ground) played a significant role in increasing or decreasing the resource use efficiency of intercrop species, e. g., increasing the inter-row spacing from 50 to 60 cm enhanced the radiation use efficiency of soybean by 15% and maize by 4%^26^. Although, in this study, we did not measure the effect of intercropping on water use of intercrop species in MSR; however, in a previous study (Rahman et al., 2017), it has been confirmed that maize and soybean utilized water more efficiently in medium-strips compared to the maize and soybean plants in narrow-strips in MSR^4^. Similarly, the inter-row spacing of 60 cm in MSR improved the uptake of nitrogen (by 25%), phosphorus (by 33%), and potassium (by 24%) in soybean compared to the inter-row spacing of 40 cm in MSR through the efficient exploitation of the biological potential for nutrient acquisition^22^. In wheat-fababean intercropping system, wheat roots uptake more nitrogen than faba bean from the rhizosphere and decrease the availability of nitrogen for fababean, which increased the percentage of nitrogen fixation in fababean^36^. All these findings revealed that the intercrop species achieved higher values of LER when they planted at an appropriate (e. g., a strip width of two meters) inter-row spacing in intercropping systems, where both crops can facilitate and complement each other in a positive way^23,32^. Moreover, the present results indicate that the higher contributions of soybean yield to MSR yield in T_2_ can be attributed to below-ground (nutrients and water) and above-ground (sunlight) complementary resource use between intercropped species in MSR; because T_2_ compared to T_1_ and T_3_ substantially enhanced the competitive ability and yield of soybean without negatively affecting the competitive ability and yield of maize^23^. In previous studies, researchers have tested the strip width of two meters or less than two meters, and they concluded that the intercropping systems achieved the highest LER values in two meters or less than two meters strip^8,13,21,37–39^. Consistent with all these reports, the results of our findings indicate that the strips of two meters (T_2_) in MSR have an advantage in utilizing the resources (i. e., light and land) more efficiently than narrow-strips (T_1_) and wider-strips (T_3_). Nevertheless, the strips of two meters wide in intercropping systems are still narrow compared with that of machinery operated by farmers in large scale mechanized agriculture. Consequently, with the current large machinery, it is not easy to obtain the claimed advantages and benefits of intercropping in strips of two meters or less. Taken together, these results emphasize the previously specified issues that intercropping systems will become less attractive to farmers, especially in developing countries, because they want to replace the labor with machinery. Therefore, we need to bring significant changes in the current farm machinery, which can be operated in strips of two meters in intercropping and relay intercropping systems.

### Economic feasibility

A recent case study on the effects of strip width management in relay intercropping systems revealed that the inter-row spacing in relay intercropping systems contributed substantially to produce high crop yields and LER, especially in systems combining C4 and C3 species^11^. Their simulations showed that the relay intercropping systems with narrow-strips (strips of two meters or less) achieved high LER, and the benefits of relay intercropping systems decreased with wider-strips. Similarly, in our study, the highest LER and net income were obtained in treatment T_2_ compared to T_1_ and T_3_ treatments. This improvement in LER and net income were mainly attributed to higher soybean yield with maintained maize yield, which ultimately increased the total LER and net income of MSR. Furthermore, increasing the distance between the rows of soybean and maize from 50 to 70 cm resulted in a significant decline in LER of MSR, indicating that as the strips become wider, the plant to plant distance within maize and soybean plants reduced, which increased the intra-specific competition for resources^35^. Besides, wider strips in MSR progressively resemble with narrow parcel alternations of sole maize and sole soybean. Therefore, the intercropping advantages and benefits start to diminish with the increase in strip widths^12^, and the complementarity in using the resources between maize and soybean in MSR becomes less likely to occur^11^.

Unfavorable environmental conditions during the life period of crops threatened the yield stability of crops, and yield stability is the main index to consider when judging the value of planting pattern^40^. Relay intercropping systems have greater yield stability compared to sole cropping systems^13^, especially in those regions where the growing seasons are too short for double cropping (e. g., Chongqing, Gansu, and Sichuan in China). Similarly, in this study, the average values of LER in T_1_, T_2_, and T_3_ are 1.53, 1.76, and 1.44, respectively, which indicates that 44% to 76% more land will be needed by sole soybean and sole maize to equal the yield of MSR. Besides, compared to SM and SS, treatments T_1_, T_2_, and T_3_ increased the net income of MSR by 11% and 9%, 98% and 94%, and 27% and 24%, respectively. These results of LER and net income clearly shows the higher yield stability, land advantages, and net income of maize/soybean relay intercropping systems over sole cropping systems under the prevailing conditions. Overall, intercropping systems remains an interesting and better option to obtain high intercrop yields with high utilization of available resources (light and land). However, for the sustainable production of cereals and legumes, we need to select the optimum inter-row spacing in MSR. These results also provide an example for obtaining high crop yields, intercropping advantages, and net income in other countries of the world, especially in developing countries (China, and Pakistan).

## Conclusion

Advantages of maize/soybean relay intercropping system in terms of the competitive ability of intercrop species and land equivalent ratio are maximum with the strip width of two meters. Economic benefits of an increased soybean grain yield with maintained maize yield are likely to be worthwhile only with the use of medium-strips in maize/soybean relay intercropping system. Wider strips significantly decreased the grain yield and net income of the maize/soybean relay intercropping system, which means that the use of wider strips in maize/soybean relay intercropping system will reduce the advantages and benefits of the intercropping system over sole cropping systems. Furthermore, small farm machinery is required to attain the maximum benefits of relay intercropping systems; without resolving the machine issue, we cannot achieve the advantages and benefits of intercropping systems.

## Materials and methods

### Experiment site

This field study was carried out at the Research Farm of the Sichuan Agricultural University in Ya’an (29°59′ N, 103°00′ E), Sichuan of southwest China from 2012 to 2013. The climate of this region is subtropical humid, with an average temperature of 16.2 °C. The frost-free period lasts approximately 300 days, with an average rainfall of 1200 mm. Weather data during the experimental seasons are shown in Table 8. The soil is purple clay loam, with a pH of 6.7, soil organic matter of 30.6 g/kg, available N of 65.5 mg/kg, available P of 18.9 mg/kg, available K of 97.6 mg/kg in the top 30 cm soil layer.

**Table 8:** Average temperature (T), rainy days, rainfall, humidity, cloud, and sun hours from March to November in cropping seasons of 2012 and 2013.

Month	Years											
	2012						2013					
	Average T (°C)	Rainy Days	Rainfall (mm)	Humidity (%)	Cloud (%)	Sun Hours	Average T (°C)	Rainy Days	Rainfall (mm)	Humidity (%)	Cloud (%)	Sun Hours
March	10	13	13	69	37	259	15	6	9	54	25	297
April	17	9	14	60	24	290	16	13	32	64	34	259
May	20	17	34	66	37	291	20	17	34	61	32	300
June	22	15	20	68	38	338	24	14	20	66	31	351
July	23	25	73	75	40	334	24	27	107	76	35	343
August	24	20	50	71	29	295	25	13	60	68	19	311
September	19	20	37	75	43	254	19	20	31	77	42	259
October	15	20	24	76	47	252	17	12	16	67	36	268
November	9	15	8	81	45	223	11	14	18	80	44	239

### Experimental design

**Figure 2:**
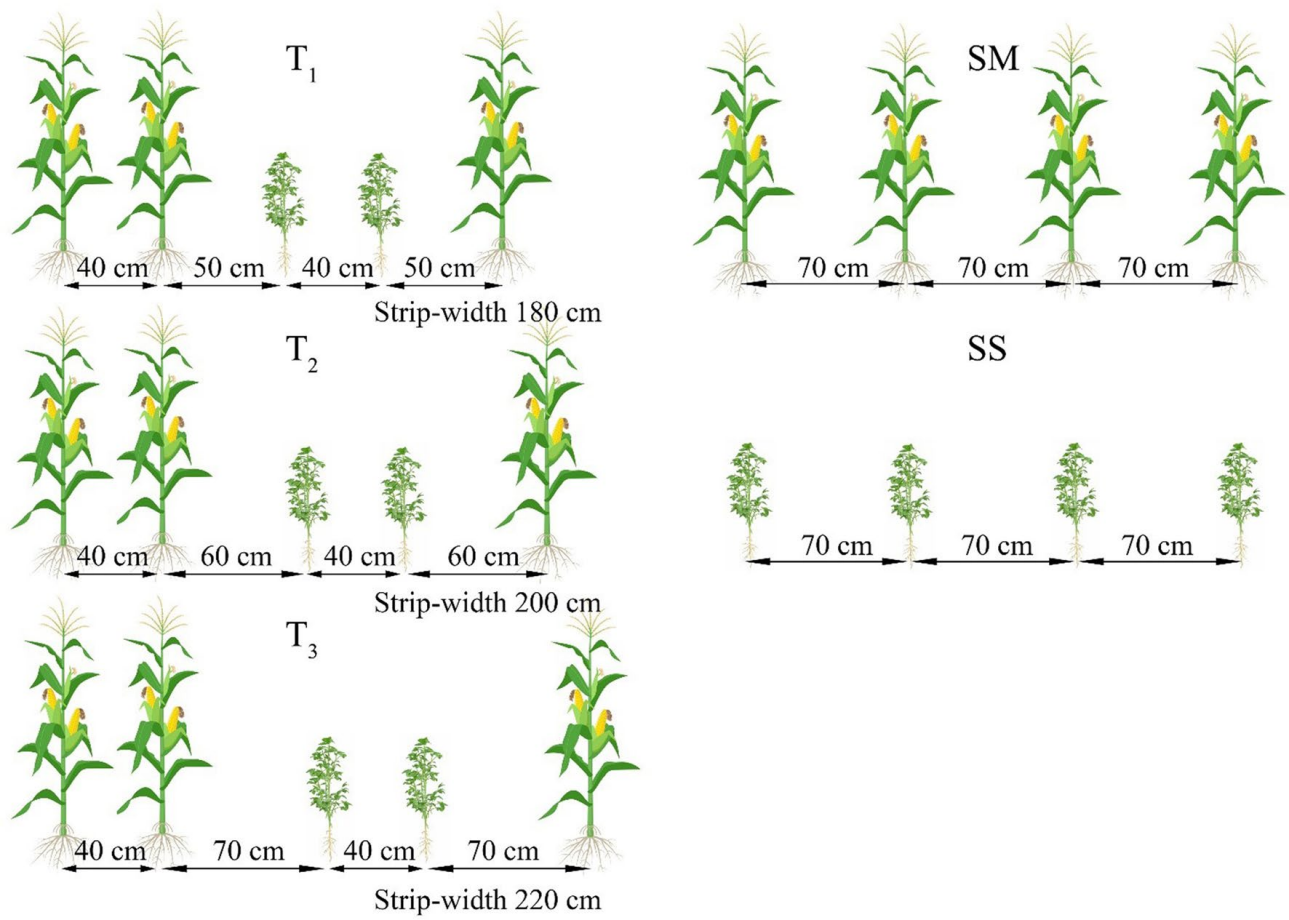
Schematic presentation of the maize soybean relay intercropping system (MSR) as affected by different strip width treatments from 2012 to 2013. In MSR, two rows of soybean were relay-intercropped with two rows of maize after the 60 ± 5 days of maize sowing. Treatment codes represent T_1_ (narrow-strips), T_2_ (medium-strips), T_3_ (wider-strips), SM (sole-maize), and SS (sole soybean).

**Figure 3:**
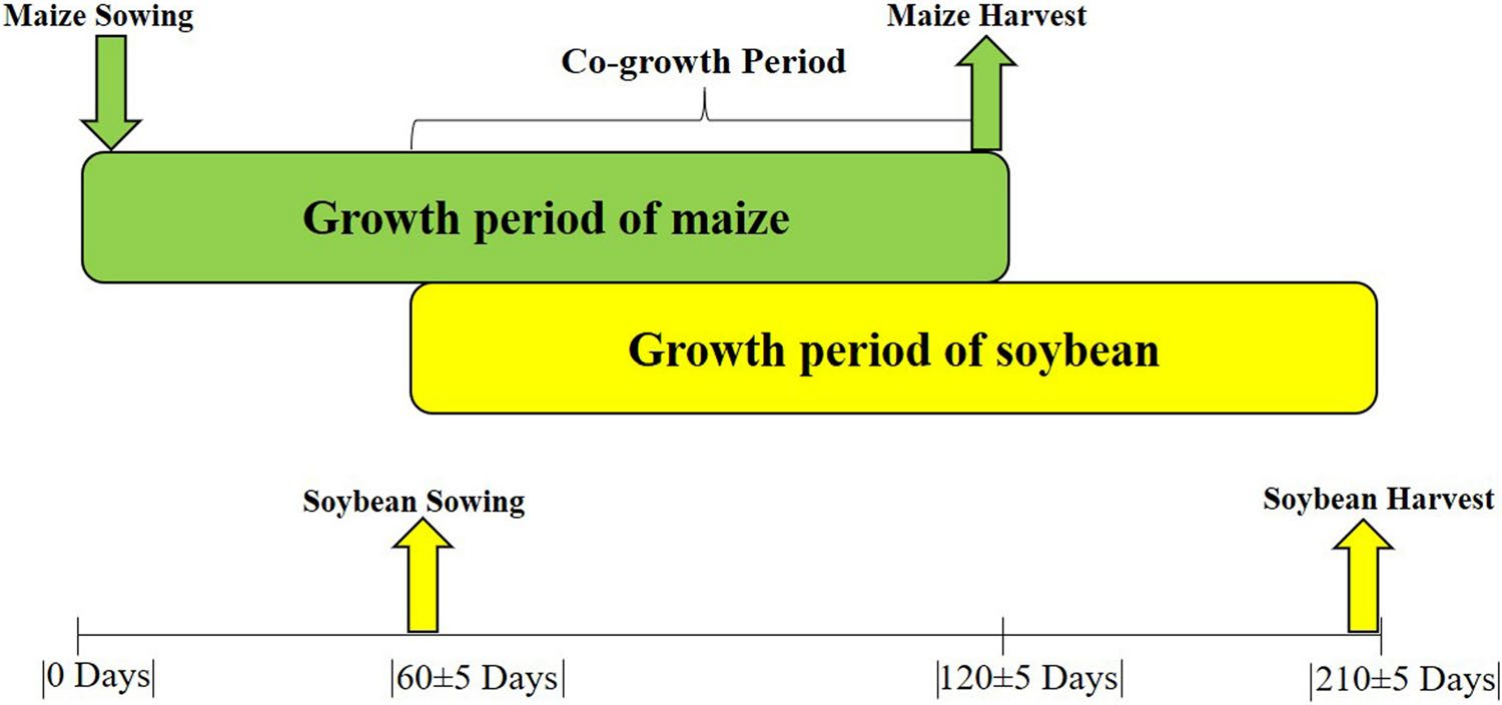
Illustration of the growth period of maize soybean relay intercropping system (MSR). The upper-bar represents the growing period of the maize crop (120 ± 5 days, first sown relay-crop), and the lower-bar shows the growing period of the soybean crop (150 ± 5 days, second sown relay-crop). The co-growth period (60 ± 5 days) is defined as the proportion of the total system growth period (210 ± 5 Days) that both relay-crops grow together.

The soybean variety (Gongxuan-1) and the maize variety (Chuandan-418) were used for this study. This study consisted of five different treatments with three replications (Fig. 2). The different cropping systems included SM (sole-maize with a row spacing of 70 cm), SS (sole soybean with a row spacing of 70 cm), and MSR (two rows of soybean were relay-intercropped with two rows of maize after the 60 ± 5 days of maize sowing). In this relay-intercropping system, three different inter-row spacing treatments in MSR are described as follows: T_1_ (narrow-strips, row spacing for soybean and maize rows was 40 cm, and the inter-row spacing between soybean and maize was 50 cm with a total strip width of 180 cm), T_2_ (medium-strips, row spacing for soybean and maize rows was 40 cm, and the inter-row spacing between soybean and maize was 60 cm with a total strip width of 200 cm), and T_3_ (wide-strips, row spacing for soybean and maize rows was 40 cm, and the inter-row spacing between soybean and maize was 70 cm with a total strip width of 220 cm). Every experimental plot consisted of four strips of soybean and maize in MSR. The size of each plot in T_1_, T_2_, and T_3_ was 43.2 m^2^ (7.2 m × 6 m), 48 m^2^ (8 m × 6 m), and 52.8 m^2^ (8.8 m × 6 m), respectively. The plant population of 1,000,000 plants per hectare for soybean was maintained uniform in SS and MSR, and 60,000 plants per hectare were kept in SM and MSR. Plant to plant spacing of 24 cm, 19 cm, 17 cm, and 15 cm for maize was maintained within each of maize in SM, T_1_, T_2_, and T_3_, respectively. For soybean, the plant to plant spacing of 14 cm, 11 cm, 10 cm, and 9 cm was kept within each row of soybean in SS, T_1_, T_2_, and T_3_, respectively. Maize was sown on March 28, 2012, and April 4, 2013, and soybean was sown on June 13 of each year. Maize was harvested on August 8, 2012, and August 2, 2013. Soybean was harvested on October 29, 2012, and October 28, 2013. In the current study, the total growth period of maize and soybean in MSR was 210 ± 5 days, and the vegetative period of soybean and the reproductive period of maize overlap with a phase of 50 ± 5 days between the maize harvesting and soybean sowing (Fig. 3). Before sowing, for maize, basal N at 135 kg ha^−1^ as urea, P at 40 kg ha^−1^ as calcium superphosphate, and K at 10 kg ha^−1^ as potassium sulfate was used in all treatments. For soybean, basal N at 75 kg ha^−1^ as urea, P at 40 kg ha^−1^ as calcium superphosphate, and K at 4 kg ha^−1^ as potassium sulfate were applied in all treatments. At the V_6_ stage of maize and R_1_ stage of soybean, basal N at 135 and 75 kg ha^−1^ as urea was applied to soybean and maize in all treatments, respectively. Weeds were controlled manually.

### Measurements

#### Photosynthetically active radiation transmittance

The photosynthetically active radiation (PAR) was measured at the fifth trifoliate stage (V5), seventh trifoliate stage (V7), and flower initiation stage (R1) of soybean because all these stages come under the co-growth period of MSR. For this purpose, the flux intensity of PAR above the soybean canopy was measured at a 10-s interval using LI-191SA quantum sensors (LI-191SA, LI-COR Inc., Lincoln, NE, USA) with an LI-1400 data logger with three replications for each plot. All quantum sensors were placed on the horizontal arm of an observation scaffold, which was 5 cm higher than the soybean canopies. All measurements were collected between 11:30 h and 12:30 h on a clear day, to minimize the external effects of atmospheric conditions. The PAR-transmittance was determined according to the previously published methods^41^.

1 $$PAR Transmittance \left(\%\right)= \frac{PARs}{PARm} \times 100$$

where PARs and PARm are the PAR at the top of soybean and maize top, respectively.

#### Leaf area index and dry matter

The leaf area index of soybean was measured at V5, V7, and R1. For this purpose, ten soybean plants were collected from every treatment. The leaf area of every single leaf was determined by multiplying the leaf length by the greatest leaf width by the crop-specific co-efficient factors of 0.75 for soybean^4^. Then, the leaf area index of soybean was determined using the previously published methods^42^. After the measurement of leaf area index, all harvested soybean samples were kept in an oven for one h at 80 °C to destroy the fresh organs of soybean plants and then dried at 65 °C until constant weight achieved for dry matter (g plant^−1^) analysis.

#### Photosynthesis

The photosynthetic characteristics, i. e., photosynthetic-rate (*Pn*), transpiration-rate (*Tr*), stomatal conductance (*Gs*), and intercellular CO_2_ concentration (*Ci*) of soybean leaves were determined using a portable photosynthesis system (Li-6400, LI-COR Inc., Lincoln, NE, USA). We measured the photosynthetic characteristics of soybean leaves at V5, V7, and R1. In all plots, three fully developed trifoliate leaves were selected to determine the photosynthetic characteristics of soybean plants. All photosynthetic measurements were taken from 11:00 to 12:00 h under the constant carbon dioxide concentration of 400 µmol mol^−1^^43^.

#### Grain yield and competition parameters

At soybean and maize maturity, 40 soybean plants and 24 maize ears were collected from each plot of every replication. After that, all obtained samples were sun-dried for the next ten days. After drying, soybean and maize samples were manually threshed and determined the grain number (plant^−1^), grain weight (mg), and grain yield (kg ha^−1^) of soybean and maize. Furthermore, the total grain yield (kg ha^−1^) of maize/soybean relay intercropping system was calculated by the summation of maize and soybean grain yields in T_1_, T_2_, T_3_ under MSR.

The land equivalent ratio (LER) was used to determine the land use advantage of soybean and maize provided relay intercropping systems. LER was calculated as follows^44^:

2 $$\mathrm{Partial\, land\, equivalent\, ratio\, of\, maize }\left(\mathrm{pLERm}\right)=\frac{SYim}{SYsm}$$

3 $$\mathrm{Partial\, land\, equivalent\, ratio\, of\, soybean }\left(\mathrm{pLERs}\right)=\frac{SYis}{SYss}$$

4 $$\mathrm{Total\, land\, equivalent\, ratio }\left(\mathrm{LER}\right)=pLERm+pLERs$$

where S*Y*_*im*_ and S*Y*_*sm*_ are maize grain yields in MSR and SM, respectively, and S*Y*_*is*_ and S*Y*_*ss*_ are soybean grain yields under MSR and SS, respectively. The competition ratio (CR) was determined to measure the competitive ability of soybean and maize in MSR. CR was calculated as follows:

5 $$CRm=\frac{LERm}{LERs}\times \frac{Asr}{Amr}$$

6 $$CRs=\frac{LERs}{LERm}\times \frac{Amr}{Asr}$$

where Asr and Amr are the sown proportion area of soybean and maize in MSR, and LERs and LERm are the partial LER values of soybean and maize, respectively^45^.

#### Economic analysis

An economic analysis was done using partial budgeting to assess the economic viability of different treatments for soybean and maize production in MSR. Total expenses for the production of maize included land rent, seedbed preparation, cost of maize seeds, cost of applied fertilizer (N, P, and K), thinning and weeding, harvesting, and threshing of soybean and maize crops were estimated based on local rates. Gross income was calculated by multiplying the measured yields with the local market prices of soybean and maize in 2012 and 2013. Net income (NI) was determined by subtracting all expenses from the gross income^46^.

### Statistical analysis

The effects of different inter-row spacing treatments on the PAR-transmittance, leaf area index, dry matter, photosynthesis, yield and yield components, land equivalent ratios, and competition ratios of soybean and maize were analyzed using Statistix 8.1. Mean values were expressed as the least significant difference. All the means were compared at a 5% probability level.

## Data Availability

The datasets generated during and/or analyzed during the current study are available from the corresponding author on reasonable request.
